# Military veteran mortality following a survived suicide attempt

**DOI:** 10.1186/1471-2458-11-374

**Published:** 2011-05-23

**Authors:** Janet Weiner, Therese S Richmond, Joseph Conigliaro, Douglas J Wiebe

**Affiliations:** 1 Leonard Davis Institute of Health Economics, University of Pennsylvania, Philadelphia, PA, USA; 2 Behavioral and Health Systems Department, School of Nursing, University of Pennsylvania, Philadelphia, PA, USA; 3 NYU-HHC Clinical and Translational Science Institute and Division of General Internal Medicine, New York University School of Medicine, New York, NY, USA; 4 Department of Biostatistics and Epidemiology, University of Pennsylvania, 902 Blockley Hall, 423 Guardian Drive, Philadelphia, PA, 19104 USA

## Abstract

**Background:**

Suicide is a global public health problem. Recently in the U.S., much attention has been given to preventing suicide and other premature mortality in veterans returning from Iraq and Afghanistan. A strong predictor of suicide is a past suicide attempt, and suicide attempters have multiple physical and mental comorbidities that put them at risk for additional causes of death. We examined mortality among U.S. military veterans after hospitalization for attempted suicide.

**Methods:**

A retrospective cohort study was conducted with all military veterans receiving inpatient treatment during 1993-1998 at United States Veterans Affairs (VA) medical facilities following a suicide attempt. Deaths occurring during 1993-2002, the most recent available year at the time, were identified through VA Beneficiary and Records Locator System data and National Death Index data. Mortality data for the general U.S. adult population were also obtained from the National Center for Health Statistics. Comparisons within the veteran cohort, between genders, and against the U.S. population were conducted with descriptive statistics and standardized mortality ratios. The actuarial method was used estimate the proportion of veterans in the cohort we expect would have survived through 2002 had they experienced the same rate of death that occurred over the study period in the U.S. population having the age and sex characteristics.

**Results:**

During 1993-1998, 10,163 veterans were treated and discharged at a VA medical center after a suicide attempt (mean age = 44 years; 91% male). There was a high prevalence of diagnosed alcohol disorder or abuse (31.8%), drug dependence or abuse (21.8%), psychoses (21.2%), depression (18.5%), and hypertension (14.2%). A total of 1,836 (18.1%) veterans died during follow up (2,941.4/100,000 person years). The cumulative survival probability after 10 years was 78.0% (95% CI = 72.9, 83.1). Hence the 10-year cumulative mortality risk was 22.0%, which was 3.0 times greater than expected. The leading causes overall were heart disease (20.2%), suicide (13.1%), and unintentional injury (12.7%). Whereas suicide was the ninth leading cause of death in the U.S. population overall (1.8%) during the study period, suicide was the leading and second leading cause among women (25.0%) and men (12.7%) in the cohort, respectively.

**Conclusions:**

Veterans who have attempted suicide face elevated risks of all-cause mortality with suicide being prominent. This represents an important population for prevention activities.

## Background

Suicide is a stubbornly resistant problem in many parts of the globe. The World Health Organization estimates that more than 1 million people die by their own hands each year, a global rate of 16 per 100,000 people[1]. In 2007, more than 34,000 people died from suicide in the U.S., a population rate of 11 per 100,000[2]. Suicide risks differ by age and gender. In the U.S., men aged 20 and over have suicide rates of more than 20 per 100,000. White men aged 65 and over are at even higher risk, with the very highest rates among white men aged 85 and over.

Many military veterans are in, and age into, risk categories for suicide. In the U.S. increasing attention is being paid to suicides among active-duty personnel and veterans. This concern is especially acute given the large numbers of returning veterans from conflicts in Iraq and Afghanistan. Recently, the Veterans Health Administration and media outlets reported that the rate of suicide among younger veterans had jumped by 26% from 2005 to 2007[3].

The question of whether veterans are at greater risk for suicide or other premature mortality has not been answered definitively. In general, "the healthy soldier effect" might be expected to lead to a decreased risk of mortality, because military populations, having passed physical examinations, are healthier than the general population. A recent systematic review of studies in the U.S., U.K. and Australia found a reduction of mortality of 10% to 25% compared to the general population, depending on the cause of death studied and the period of follow up[4].

However, the healthy soldier effect does not seem to protect veterans from suicide mortality. Depending on the veteran population studied, the suicide risk is equal to or greater than in the general population. A recent cohort study of 500,000 middle-aged and elderly men found that veteran status per se did not confer an increased risk of death from suicide,[5] contradicting another large prospective cohort study that found a twofold increase in the risk for suicide among veterans[6]. One study of the subpopulation of veterans served by the Veterans Health Administration in 2001 found a 66% greater risk for suicide than in the general population[7]. Two recent review articles have concluded that veterans of the Vietnam and 1991 Gulf War era are at no greater risk, although subpopulations of Vietnam veterans, such as those deployed to Vietnam, those hospitalized for a combat wound, and those with posttraumatic stress disorder (PTSD) have higher rates of suicide[8,9]. More recent veterans of Operation Enduring Freedom (OEF) and Operation Iraqi Freedom (OIF) display a healthy solider effect for all-cause mortality, but seem to have a suicide risk that is higher than that among previous veteran cohorts, and higher than that among the general population based on follow-up through 2007[10,11]. Former active duty OEF/OIF veterans and those with PTSD had a 33% greater risk for suicide.

The U.S. Department of Veterans Affairs (VA) has made suicide prevention a national priority. In 2007, it founded a national suicide prevention hotline[12] to ensure veterans have free, 24/7 access to trained counselors[13]. And in January 2010, the VA hosted a suicide prevention conference with the goals of raising awareness of community-based tools, programs, best practices for suicide prevention and resilience building available to families, veterans, active duty personnel, clinicians, and researchers[14].

The efficiency of prevention efforts can be enhanced by focusing on populations at greatest risk. A link between mental health and suicide among veterans is well established[15-19]. With the nearly 200,000 military personnel involved in combat operations in Iraq and Afghanistan that are currently underway,[20] the risk of suicide that stems from depression and post traumatic stress disorder (PTSD) is of increasing concern[18]. One of the strongest risk factors for suicide, however, is a past suicide attempt itself[21-24]. Studies, mostly from the U.K. and Northern Europe, have found that the risk of suicide after an attempt is about 5-10% in follow-up of 5-35 years[21,22,25-28]. Little research has been conducted to follow suicide attempters in the U.S., and little is known about suicide attempters who are also veterans. One study involved a case series of 137 veterans at a VA medical facility and found that after seven years, 20 had died, 10 of whom had committed suicide[29]. The study was too small to draw conclusions about their long-term mortality risk. To inform current efforts around veterans' health, we take a look back at suicide attempters cared for at VA hospitals. We follow a large cohort and report on subsequent rates of suicide as well as other causes of death.

The goals of this study were to determine the incidence, rate, and causes of mortality among military veterans in the U.S. during the years after having attempted suicide, and to determine how the mortality rate in this veteran cohort compared to that of the U.S. population.

## Methods

### Study Design

A retrospective cohort study was conducted in which all U.S. military veterans treated at VA medical facilities during 1993-1998 after attempting suicide were followed through the end of 2002 to identify mortality from all causes. The incidence, rate, and causes of mortality were examined. To gauge the relative magnitude of mortality in the cohort, the rate of mortality that would have been expected in the cohort had death occurred at the same rate that occurred in the age- and sex-matched population of the U.S. population during the study period was calculated for comparison. The project was approved by the Institutional Review Boards of the University of Pennsylvania and the Department of Veterans Affairs Medical Research Center and Development Service.

### Data Sources

The cohort of veterans receiving medical treatment during 1993-1998 was identified through the Patient Treatment File (PTF), an automated discharge record of all episodes of VA inpatient care. The PTF is part of the VA's National Patient Care Database and captures 100% of inpatient encounters at VA medical facilities and contains administrative, demographic, and diagnostic data for each visit. The sample studied here consisted of all patients discharged from 1993-1998 with a primary or secondary diagnosis of suicide attempt (E-codes 950-959 of the *International Classification of Diseases*, Ninth Revision, ICD-9). Instances when an individual had multiple hospitalizations with a diagnosis of suicide attempt during 1993-1998 were identified and the first instance was used as the individual's entry into the cohort.

The variables obtained from the PTF for each veteran in the cohort were age, gender, race, marital status, zip code of residence, medical and psychiatric discharge diagnoses, period of military service, VA eligibility status (e.g., percent service-connected disability), and service-connected exposures (e.g., prisoner of war, spinal cord injury, radiation exposure).

Deaths subsequent to a hospitalization for a suicide attempt that occurred in the cohort during 1993-2002 were identified by searching the VA Beneficiary and Records Locator System (BRLS), an administrative database of veterans who have received VA death benefits, and identifying decedents who linked to patient records in the PTF file. The BRLS enables identification of veterans who have died but does not specify cause of death. Cause of death was subsequently obtained by a search of the National Death Index (NDI) of the National Center for Health Statistics. This is a national archive of death certificates filed in State vital statistics offices; 2002 was the most recent year available. Causes of death in the NDI were classified according to the ICD-9 prior to 1999 and the ICD-10 thereafter. The utility of determining veterans' causes of death with this two-step approach with the BRLS and NDI, rather than searching the NDI from patient records directly, has been previously established[30]. Instances of multiple matches were resolved through established procedures[31].

Data to determine the leading causes of death that occurred in the U.S. overall adult population 18 years or older during 1993-2002, and the mechanisms of injury for suicide deaths, were obtained from the National Center for Health Statistics[32]. Five-year abridged life tables from 1993 and 1998 for the U.S. were obtained to generate an estimate of how mortality in veterans over the years following hospitalization for attempted suicide compared to that we expect would have occurred in the age- and sex-matched U.S. population[33,34].

### Analysis

Descriptive statistics were generated to describe the veteran cohort in terms of demographics, medical diagnoses, and causes of mortality. Crude rates of mortality were calculated for the cohort and standardized mortality rates and rate ratios were calculated for male and female veterans to make comparisons between the two[35]. The denominators for the rates were calculated as the number of person-time days that accrued between when a veteran was admitted to a hospital following a suicide attempt until the date of death or, for those who did not die during follow up, until December 31, 2002. Standardized mortality rate ratios were also used to compare veterans who did versus did not return during the follow-up period to a VA medical facility for a subsequent treatment visit with the discharge diagnosis of suicide attempt. Age adjustment was performed in all mortality rate ratios using 10-year groupings. The baseline characteristics and cause of death information (i.e., ICD-9 E-codes) is presented overall and also by sex, and the prevalence of firearm use in male and female suicides was compared with a chi-square test. Lastly, we estimated the proportion of veterans in the cohort we expect would have survived through 2002 had they experienced the same rate of death that occurred over the study period in the U.S. population having the age and sex characteristics. This was conducted by sectioning the cohort by sex and by five-year age groupings and calculating the proportion of veterans surviving between the time of their index VA medical center visit through 2002 using the actuarial method[36]. Then, the proportion of the U.S. population that died during 1993-1997 and during 1998-2002 was applied in sex-specific, five-year age groupings to the distribution of the veteran cohort.

## Results

A total of 10,163 veterans were treated and discharged after a suicide attempt at a VA medical facility during the 1993-1998 study period. The number of veterans treated each year ranged from 1,981 in 1993 to 1,547 in 1998, and decreased each year by an average of 4.4%.

Characteristics of the cohort are shown in Table 1. Ages ranged from 15 to 101 years with a mean of 44.0 years. Most (91.2%) were male. Veterans of the Vietnam War comprised the majority of the cohort (51.2%). The most common co-morbid medical conditions were alcohol disorder or abuse (31.8%, ICD-9 303, 305.0-.03), drug dependence or abuse (21.8%, ICD-9 304, 305.90-93), psychoses (21.2%, ICD-9 290-299), depression (18.5%, ICD-9 206.2-.3, 311), hypertension (14.2%, ICD-9 401-405), chronic pulmonary disease (7.2%, ICD-9 416), neurological disorders (6.4%, ICD-9 320-389), and diabetes (5.8%, ICD-9 250).

**Table 1:** Characteristics of U.S. veteran patients treated after a suicide attempt, 1993-1998

	All patients	Male	Female
No.	10,163	9,273	890
%	100	91.2	8.8
Age, years			
Mean (SD)	44.0 (10.8)	44.6 (10.7)	38.1 (9.6)
Median	43	44	38
Age distribution, %			
<29	6.7	5.8	16.4
30-39	26.8	25.3	42.8
40-49	44.6	45.8	32.7
50-59	13.0	13.8	4.6
60-69	5.3	5.6	2.0
70-79	3.1	3.3	1.4
80	0.5	0.6	0.1
Race/ethnicity, %			
White	70.4	70.2	72.6
African American	20.3	20.4	19.6
Hispanic white	6.0	6.2	3.5
Hispanic African American	0.5	0.5	0.2
American Indian	0.7	0.8	0.6
Asian American	0.3	0.3	0.3
Unknown	1.7	1.6	3.3
Marital status, %			
Divorced	35.3	35.8	30.2
Married	28.3	28.4	27.0
Never married/single	20.9	20.5	24.8
Single	12.3	12.2	13.3
Widowed	2.6	2.6	3.6
Unknown	0.6	0.6	1.1
Period of service, %			
WWI	0.0	0.0	0.0
WWII	4.0	4.2	1.7
Pre-Korea	0.2	0.2	0.0
Korea	3.9	4.2	1.2
Post-Korea	4.2	4.5	1.4
Vietnam	51.2	54.0	22.1
Post-Vietnam	27.6	25.8	46.1
Other	1.8	1.2	8.3
Storm (ACT)	0.6	0.4	1.8
Storm (RET)	6.5	5.5	17.4
Comorbidities, %			
Alcohol disorder or abuse	31.8	32.9	20.7
Drug dependence or abuse	21.8	22.6	13.5
Psychoses	21.2	21.0	22.7
Depression	18.5	18.3	20.3
Hypertension	14.2	14.9	7.2
Chronic pulmonary disease	7.2	7.3	5.6
Neurological disorders	6.4	6.5	5.5
Diabetes	5.8	6.0	3.3

SD denotes standard deviation.

Approximately one in every ten (12.4%) of the 10,163 veterans were treated for at least one subsequent incident of suicide attempt at a VA medical center during the 1993-1998 period. 1,840 medical center visits for suicide attempt were made by the 1,261 veterans in total. Most (938, 74.4%) of those who returned did so only once. However, 209 (16.6%) were seen again twice, 68 (5.4%) were seen again three times, and 46 (3.7%) were seen again four of more times with the maximum number of visits being 15.

**Figure 1:**
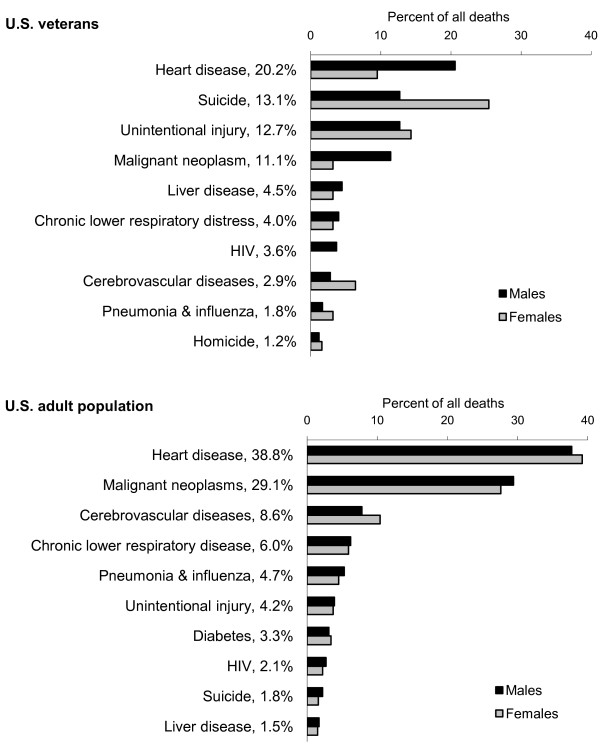
**Ten leading causes of death in 10,361 U.S. veterans and the U.S. adult population during 1993-2002**.

A total of 1,836 of the 10,163 veterans died during the 1993-2002 follow-up period (18.1%). The mortality rate, accounting for follow-up time in the veteran cohort, was 2,775.9/100,000 person years. Death certificates were successfully matched for 96.3% (1,768) of the 1,836 decedents. The ten leading causes of death in the cohort of veterans during 1993-2002 are shown in Figure 1, with percentages indicating the proportion of deaths that each cause of death accounted for among the subsamples of males and females. Over half of the deaths (57.1%) were due to four diseases - heart disease, suicide, unintentional injury, and malignant neoplasm - with suicide being the leading cause of death among females (25.0%) and the second leading cause of death among males (20.6%).

Among males, poisoning (E950.0-E952.9) and firearms (E955.0-.4) accounted for the majority suicides, with each accounting for 38% of the suicides (38.9% and 38.0%, respectively). Suffocation (E953.0-.9) accounted for 9.5%, thus together these three manners accounted for 86.6% of male suicides. These three manners accounted for 100% of the female suicides, however poisoning accounted for 50.0%, firearms accounted for 31.3%, and suffocation accounted for 18.8%. As such, the proportion of suicides committed with firearms did not differ between male and female veterans (χ^2^[1] = 0.286, p = 0.59).

A total of 18,624,180 deaths occurred in the U.S. adult population overall during the 1993-2002 span of time that the cohort of veterans was followed. Of the ten leading causes of death in the cohort of veterans, nine were also observed among the ten leading causes of death that occurred in the U.S. adult population (Figure 1). However, the order of their rankings was noticeably different. Two thirds (66.9%) of deaths in the U.S. population were due to heart disease and malignant neoplasm, and suicide ranked ninth and accounted for 1.8% of deaths. In contrast, suicide ranked second and accounted for 13.1% of deaths among veteran cohort overall. Suicide was the leading cause of death among women (25.0%) and was the second leading cause of death men (12.7%) in the veteran cohort. The tenth most common cause of death was liver disease among the U.S. population and was homicide among veterans.

The crude rate of all-cause mortality was 2,941.4/100,000 among male veterans and 1,116.6/100,000 among female veterans during the 1993-2002 period, with a standardized mortality rate ratio comparing men to women of 1.92 (95% CI = 1.38, 2.67) (Table 2). The crude rates of all-cause mortality among veterans who had at least one versus no subsequent hospitalizations at a VA medical facility with a discharge diagnosis of suicide attempt were 2712.4/100,000 and 2785.8/100,000 respectively, with a standardized mortality rate ratio indicating little difference in the rate of mortality by all causes between the two (1.13, 95% CI = 0.99, 1.30). Alternatively, the relative rate of mortality by suicide specifically was 1.47 times (95% CI = 1.05, 2.06) higher among veterans who had at least one versus no subsequent hospitalizations with a discharge diagnosis of suicide attempt, after adjustment of the crude rates (the standardized mortality ratios were 459.5/100,000 and 333.8/100,000 respectively).

**Table 2:** Age-specific mortality rates by gender among discharged VA patients over the period 1993-2002, and mortality rate ratio

		**Males**			**Females**		
**Age group**	**Deaths**	**Person years of follow-up**	Rate of death*	**Deaths**	**Person years of follow-up**	death*	**Rate ratio (RR)**
			
Under 30	33	3827.3	862.2	7	1017.3	688.1	1.25
30-39	250	16486.9	1516.4	18	2672.3	673.6	2.25
40-49	739	27929.8	2645.9	26	1902.8	1366.4	1.94
50-59	309	7624.6	4052.7	2	236.6	845.5	4.79
60-69	206	2822.3	7299.1	5	109.0	4586.8	1.59
70 and older	232	1450.4	15995.5	9	62.4	14424.1	1.11
			
Total	1769	60141.3		67	6000.4		2.63
	Crude rate = 2941.4 Standardized rate = 1953.7			Crude rate = 1116.6 Standardized rate = 1099.1			
				Standardized rate ratio = 1.92 (95% CI 1.38 to 2.67)			

Standardized rates and standardized rate ratio computed using males as the standard.

* Rate per 100,000 person years.

**Figure 2:**
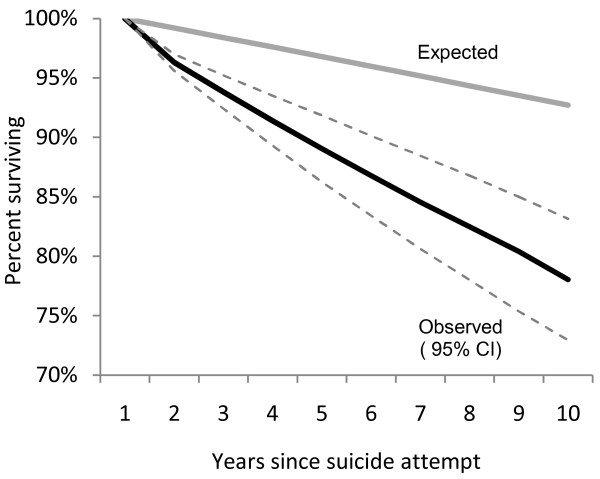
**Observed and expected survival among 10,361 U.S. veterans during years following a suicide attempt**.

The percent of veterans surviving in this cohort over the 10-year follow-up period was 78.0% (95% CI = 72.9, 83.1) (Figure 2). An estimated 92.7% would have been alive at the end of the follow-up period had the cohort experienced the same mortality rate that occurred in the U.S. population over the same period. These translate to a 22.0% observed and a 7.3% expected 10-year cumulative risk of death, and thus a risk of death in the veteran cohort that was 3.0 times greater than we expect would have occurred in the cohort had the veterans experienced the same mortality rate that occurred in the segment of the general population with the same age and sex characteristics.

## Discussion

The health and wellbeing of those who have served in the military is a priority of nations worldwide. Suicide is a prominent health concern in this large population and the prevention of suicide is possible. This study presents new information regarding veterans in the U.S. who have made a suicide attempt in the past. The 10-year risk of mortality among veterans was an estimated 3.0 times higher than would have been expected in a cohort from the overall U.S. population with the same age and sex characteristics, and was found to be largely accounted for by heart disease, suicide, unintentional injury, and malignant neoplasm. Until now, attention has been directed toward the health of military personnel after their periods of service and has been focused on raising awareness about veterans and the risk of suicide and making resources for suicide prevention available in general. The results presented here indicate that veterans who have attempted suicide represent a unique community that should be a focus of prevention efforts that extend well beyond self harm and suicide to other major chronic diseases, specifically heart disease, cancer, and unintentional injuries.

As in studies of veterans overall, comorbid physical and mental conditions were common among veterans who had been hospitalized for treatment after attempting suicide. Given our finding that non-suicide causes of mortality were elevated in veterans, it is important for clinicians to broaden their focus on optimal management of these comorbidities that contribute to early mortality. Indeed, the top comorbid conditions in the veteran cohort, such as alcohol disorder and abuse, drug dependence and abuse, and psychosis could in part explain the excess frequency of unintentional injury and subsequent mortality. Similarly, given the known contribution of depression to new and recurrent cardiovascular events,[37] and to excess mortality after myocardial infarction,[38] stroke,[39] and injury, our findings strongly indicate the need to aggressively manage depression as a comorbidity in veteran suicide attempters.

Many of the veterans that comprised the sample had multiple inpatient hospital treatments with a discharge diagnosis of suicide attempt within a span of six years. And the rate of suicide over the ensuing years was 46% higher among these veterans as compared to veterans who during the observation period had received inpatient treatment only once. This too identifies a need to aggressively manage the challenges facing veterans following an episode of self harm.

The use of firearms for suicide in the U.S. adult population overall is more common among males than females (59.6% and 33.0% respectively in 2002)[32]. But firearms were used in approximately one-third of the suicides in the cohort by male female veterans alike. The disproportionately low use of firearms in male veterans relative to males in the general population may reflect that male veterans in the cohort study who had a propensity to use a firearm to attempt suicide did use a firearm during their first attempt. Given the lethality of this method, most of those veterans would have died and thus perhaps those male veterans who made a subsequent attempt during the study period and were included in our database were those who did not have a propensity to use a firearm. Living in a home where firearms are present is a risk factor for household members to commit suicide[40-42]. Given the familiarity with and access to firearms that stems from military experience, limiting access to firearms among female and male veterans alike should be pursued as one strategy for prevention[43]. In fact in 2008, the Department of Veterans Affairs published a safety plan manual to give physicians structured guidance for how to identify veterans who were considering suicide and to take actions to reduce their risk[44]. The manual includes guidance for asking veterans about whether they have access to a gun and then making arrangements to secure the weapon.

Other prevention strategies should also to be pursued. One example is the suicide prevention program that the U.S. Air Force launched in response a suicide rate that was increasing among active Air Force personnel in the early 1990s[45]. Starting in 1996, the Air Force implemented a nationwide program that involved community agencies inside and outside the healthcare sector and focused on removing the stigma of seeking help for a mental health or psychosocial problem, enhancing understanding of mental health, and changing policies and social norms. Implementation of the program was associated with a sustained reduction in the suicide rate and a 33% reduction in suicide risk[45]. A concerted effort similarly focused on the veteran population appears warranted.

To the best of our knowledge, this is the largest follow up study of suicide attempters in the U.S. This is also the first large study of suicide attempters who are veterans. Despite the strengths that stem from having a large sample size and comprehensive method of follow-up, the study has limitations. The cohort was identified according to diagnosis codes in a VA patient data file. One data audit found that diagnosis codes were accurate for the great majority of cases in VA patient data (88%)[46]. Thus it is reasonable to expect that cohort that was obtained largely, though not perfectly, represented the patient population we aimed to study. Also, our measurement of an index, non-fatal suicide attempt is limited to those that resulted in hospitalization at a VA medical facility. It is therefore likely to be an underestimate of all suicide attempts because it does not include attempts treated through emergency departments or on an outpatient basis, nor does it include attempts that resulted in no medical treatment or hospitalizations outside the VA system. However there is evidence that the rate of cross-system use for veterans utilizing VA facilities for mental health reasons is low[47]. Our primary outcome of interest, mortality, was measured well through our strategy of identifying deaths through beneficiary records and determining causes of death subsequently through a search of the NDI[30]. Even so, it remains possible that the incidence of mortality in the study cohort is an underestimate due to unidentified deaths. Perhaps a more likely possibility is that some suicides that occurred in the cohort were classified as another cause of death. While some have suggested that efforts to avoid stigma lead to underreporting, Timmermans suggests that the challenge involved in establishing suicidal intent may be what, if anything, drives underreporting of suicide[48]. We are unable to gauge if or how often this happened in the cohort we studied. However, for most suicides there is typically adequate evidence to establish intent[48]. Moreover, a past suicide attempt is one of the criteria used to establish intent. Given that a past suicide attempt was well documented for each member of the cohort, perhaps suicides that occurred in the cohort were more accurately classified than would be suicides among the general population. Ultimately, we anticipate that misclassifications would be infrequent in the cohort and that the effect of any such instances would be to make the results presented here be an underestimate the incidence of suicide that actually occurred.

## Conclusions

This study demonstrates the importance of acknowledging the need for suicide prevention efforts for individuals at risk, and veterans who have attempted suicide are one such priority. The challenges these veterans face include risks of mortality from multiple other causes as well. This information can be used in allocating resources for developing strategies for prevention.

## Competing interests

The authors declare that they have no competing interests.

## Authors' contributions

JW conceived of the study and JW, DW, and TR obtained funding. DW conducted the analysis and all authors contributed to interpretation of the results. JW and DW drafted the manuscript and TR and JC conducted revisions to the manuscript. All authors read and approved the final manuscript.

## Pre-publication history

The pre-publication history for this paper can be accessed here:

http://www.biomedcentral.com/1471-2458/11/374/prepub
